# Headache disorders: a persistent public health challenge for the under 50s

**DOI:** 10.3389/fneur.2024.1501749

**Published:** 2024-10-23

**Authors:** Weijian Meng, Liutao Sui

**Affiliations:** ^1^Molecular Medicine Center, Tongji Hospital, Tongji Medical College, Huazhong University of Science and Technology, Wuhan, China; ^2^Department of GI Surgery, Tongji Hospital, Tongji Medical College, Huazhong University of Science and Technology, Wuhan, China; ^3^Department of Critical Care Medicine, The Affiliated Hospital of Qingdao University, Qingdao, China

**Keywords:** headache disorders, incidence, prevalence, disability-adjusted life years, prediction, global burden of disease 2021

## Abstract

**Introduction:**

Currently, neurological diseases has surpassed cardiovascular diseases as the primary cause of global disease burden. Among these, headache disorders are the most prevalent and have emerged as the main cause of disability in people under 50 years old in recent years. Since the release of GBD 2021, there has been no comprehensive systematic exposition on the burden of headache among individuals under 50 years old and a forecast for future burdens. This study aims to quantify the global, regional, and national burden of headache disorders among people under 50 from 1992 to 2021 and to predict future trends in order to provide policy makers with precise and effective epidemiological evidence.

**Methods:**

This study extracted the incidence, prevalence, and DALYs data related to headache disorders in the 5–50 age group from the GBD 2021. After age-standardizing the data, we used joinpoint regression analysis and health inequity analysis to analyze the burden and temporal trend of headache disorders and predicted the future disease burden and changes based on the age-period-cohort model.

**Results:**

By 2021, the case number of global incidence, prevalence and DALYs have increased by 35, 39, and 41%, respectively, over the past 30 years. The incidence and prevalence of tension-type headache (TTH) are significantly higher than those of migraine, but migraine causes greater health burdens. The burden is higher for female than for male. In terms of age, youth aged 25–29 years had the highest incidence in 2021, with an age-standardized rate (ASR) of 13,454.64 (95%CI, 9,546.96–18,361.36) per 100,000 population. Nationally, the highest ASR of incidence and prevalence are found in Norway, and the most damaging to health is found in Belgium. Among the five sociodemographic index (SDI) categories, the middle SDI has the highest number of cases (190 million in 2021). It is worth noting that the forecast shows that by 2046 the three indicators will reach 680 million, 2.33 billion, and 41 million, respectively, indicating that the burden of headache disorders in this age group will continue to persist.

**Conclusion:**

Globally, the burden of headache disorders in people under 50 years of age remains significant and has not improved over the past 30 years, especially in regions with high SDI. Headache problems will continue to pose a serious public health challenge for this age group for some time to come. This study reveals the burden and distribution of headache disorders in this age group, providing important basis for governments and policymakers to accurately and effectively allocate health care resources, strengthen prevention and management strategies, and respond to this global health problem.

## 1 Introduction

Headache is a temporary or permanent functional disorder of the central nervous system that is a common health problem experienced by many people throughout their lives (1, 2). Research shows that the lifetime prevalence of headache in adults is as high as 52%, making it a significant burden on individuals and society (3). Repeated attacks of headache and the constant worry about a recurrence can affect family, society, and employment, and long-term struggle with chronic headache may also increase other health risks. Due to its ability to cause disability and incur high financial costs, headache has become a serious public health challenge (4–7). The study of Waliszewska-Prosol and Echiverri et al. indicate that headaches typically onset during childhood and adolescence, rather than in adulthood as is commonly assumed (8, 9). Steiner et al. showed that headache disorders are the third cause of global disability, expressed as years lived with disability (YLDs), in the first in the 15–49 age range (10). Based on these findings, we believe that it is important to explore the problem of headache in people under 50 years of age. As the aging of the global population intensifies, this group will become the core force for addressing population structure changes. Maintaining the health of this group is of paramount importance for reducing social and economic burden and promoting social sustainable development. However, headache stigma leads to discrimination in the workplace, healthcare settings and personal relationships, as well as feelings of shame, guilt and isolation, leading to greater avoidance of seeking medical care and treatment (11). At the same time, disparities in health care, treatment opportunities, and drug availability pose important issues that need to be addressed in headache medicine (12). The unequal distribution of health resources between different regions or countries makes it difficult for certain groups to access timely and effective treatment. This imbalance not only limits the ability of patients to access appropriate care, but can also lead to worsening conditions, thereby increasing the overall socio-economic burden. Therefore, the importance of enhancing awareness of this public health problem is particularly prominent.

The GBD 2021 provides us with the latest detailed epidemiological data to help us quantify health levels and disease trends. Recent studies have only used GBD 2021 to report the burden of migraine in childbearing age women and new insights into childhood headaches (13, 14). But there is still a lack of in-depth and comprehensive discussion on the burden of headache disorders in people under 50s and future trends. This study uses the latest headache disorders data from GBD 2021 to conduct in-depth analysis of the incidence, prevalence, and DALYs of headache disorders globally, regionally, and nationally from 1992 to 2021, and predicts the trends of headache disorders in the next 25 years. We hope that this paper will raise awareness of headache disorders, provide the scientific basis for mitigating their health challenges, and assist countries and regions in formulating public health policies to reduce the increasing financial and health burden.

## 2 Materials and methods

### 2.1 Data source

This study is based on data from GBD 2021, which provides the most recent epidemiological data estimates of the burden of disease and injury for 21 GBD regions and 204 countries and territories from 1992 to 2021. All this data is accessible for free access through the Global Health Data Exchange query tool.^1^ The GBD 2021 disease and injury burden analysis estimated years lived with incidence, prevalence, years lived with disability (YLDs), years of life lost (YLLs) and disability-adjusted life-years (DALYs) for 371 diseases and injuries using 100,983 data sources (15). The SDI serves as an indicator of a country’s or region’s developmental status (16). The 204 countries and territories were classified into five SDI regions: low, low-middle, middle, high-middle, and high in the GBD 2021. The data obtained from GBD 2021 shows that the 204 countries and territories of GBD are divided into quartiles: high SDI (0.81–1), high SDI (0.71–0.81), middle SDI (0.62–0.71), middle and low SDI (0.77–0.62), and low SDI (0–0.47). This paper discusses the burden of headache disease in 5–49 years of age, excluding the age group less than 5 years of age, because data below 5 years of age are missing.

### 2.2 Case definition

In the GBD 2021, headache disorders are classified into migraine and TTH. Migraine is a disabling primary headache disorder, typically characterized by recurrent moderate or severe unilateral pulsatile headaches. The two major types are migraine without aura and migraine with aura (transient neurological symptoms). In GBD, we do not distinguish between migraine with and without aura as most epidemiological studies report on overall migraine only. The reference diagnostic criteria for migraine are from the International Classification of Headache Disorders (ICHD)-3, and when the five main criteria are met, it is migraine; TTH is characterized by a dull, non-pulsatile, diffuse, band-like (or vice-like) pain of mild to moderate intensity in the head or neck. Similar to migraine, the five diagnostic criteria of ICHD-3 must be met to be defined as TTH.

### 2.3 Jointpoint regression analysis

The joinpoint regression analysis is used to identify inflection points in the trend of disease changes (referred to as joinpoints) and calculate the annual percentage change (APC) between these inflection points, as well as the average annual percentage change (AAPC) over the entire period. First, a piecewise regression using a logarithmic linear model was conducted, and all potential junction points were established using the grid search method (GSM). The mean squared errors (MSE) for each possible scenario were calculated, and the junction point corresponding to the minimum MSE was selected. Subsequently, the determination of the optimal number of junction points in the segmented regression model was performed using Monte Carlo permutation tests. The maximum potential number of junction points was set at 5, while the minimum potential number was set at 0. Permutation testing began with the number of join points *k* = 0 and *k*_max = 5; if *k* ≠ *k*_max, then *k* = *k* + 1 for further testing until the model corresponding to *k* = *k*_max was identified as optimal. Finally, APC and AAPC quantifying trends from 1992 to 2021 were computed using the optimal model (17).

### 2.4 Analysis of health inequities

Health inequality monitoring is essential for evidence-based health planning and the enhancement of relevant policies, programs, and practices to address disparities in health distribution. In this study, we applied two standard metrics, namely the slope index of inequality and concentration index, to measure the distributive inequality of Headache disorders burden across countries. The slope index was calculated by regressing national DALYs rates across all age groups on a relative position scale associated with sociodemographic development. Additionally, the concentration index was determined by numerically integrating the area under the Lorenz concentration curve using cumulative fractions of DALYs and cumulative relative distributions of populations ranked by SDI (18, 19).

### 2.5 Prediction of disease burden

Utilizing a log-linear age–period–cohort model to predict the number and rates of incidence, prevalence, and DALYs globally from 2022 to 2046 based on recent trends. GBD 2021 incidence, prevalence and DALYs estimates for headache disorders and population data for each country were used as data inputs for the age-period-cohort model. In a typical age-period-cohort model, the interval between age and period must be equal, i.e., five-year age groups should be used with five-year calendar periods. Therefore, we divided the 5–49 age group into 9 age groups (5–9, 10–14, …, 45–49), the distance between the groups was 5 years. The period from 1992 to 2021 is divided into six consecutive five-year periods (1992–1996, 1997–2001, …, 2012–2016, 2017–2021), and then using the Nordpred software package implemented in R language to make predictions. It has been empirically validated for effectively projecting recent trends into the future by extrapolating the most recent 5-year observed periods using a power function to stabilize growth. Adjustments were made to the linear trend of the recent decade in the second, third, and fourth prediction periods by either attenuating or accentuating it by 25, 50, and 75%, respectively. Further details can be found elsewhere. To estimate the number of incident cases, prevalent cases, and DALYs counts for 2046, we calculated a weighted average of projected rates for the final two prediction periods and applied these rates to GBD 2017 population forecasts accessible globally in that year. For statistical analysis and visualizations, joinpoint regression software and R were employed. All statistical tests were two-sided with *p*-values less than 0.05 considered statistically significant (17).

### 2.6 Statistical analysis

Given the diverse age distribution and population represented in the GBD dataset, adjustments for changes in age structure are necessary. Therefore, the ASR per 100,000 population was calculated using the subsequent formula (20):


$$ASR=\frac{\Sigma_{i=1}^{A}a_{i}w_{i}}{\Sigma_{i=1}^{A}w_{i}}$$

where *a_i_* is the age specific rate and *w_i_* is the weight in the same age subgroup of the chosen reference standard population (in which *i* denotes the *i*th age class) and *A* is the upper age limit.

To quantify the changing trends in rates from 1992 to 2021, the following formula is used to calculate the AAPC (20):


$$\mathrm{AAPC}=\left\{\exp\;\left(\frac{\Sigma w_{i}b_{i}}{\Sigma w_{i\;}}\right)\;-1\right\}$$

*b_i_* is the slope coefficient for the *i*th segment with *i* indexing the segments in the desired range of years, and *w_i_* is the length of each segment in the range of years.

Data cleaning, analysis, and visualization were performed using R software (version 4.3.2) in this research. The ggplot2 package was utilized for generating visual representations, and final adjustments were made using Adobe Illustrator.

### 2.7 Limitations

Based on GBD 2021, we reveal for the first time the global burden of headache disorders in people under 50 years of age. However, this study has several limitations. Firstly, the lower standards of health care in some less developed countries may lead to misdiagnosis and underdiagnosis, thereby underestimating the burden of disease. Secondly, the data obtained from GBD relies heavily on statistical modeling methods, as GBD collaborators employ multiple statistical models in countries where raw data is lacking. Finally, the global population ratio results used in the age-standardized calculations carry confidence intervals, which call for careful interpretation of the findings and emphasize the need for further real-world studies to validate these findings.

## 3 Results

### 3.1 Global trends of headache disorders

**Figure 1:**
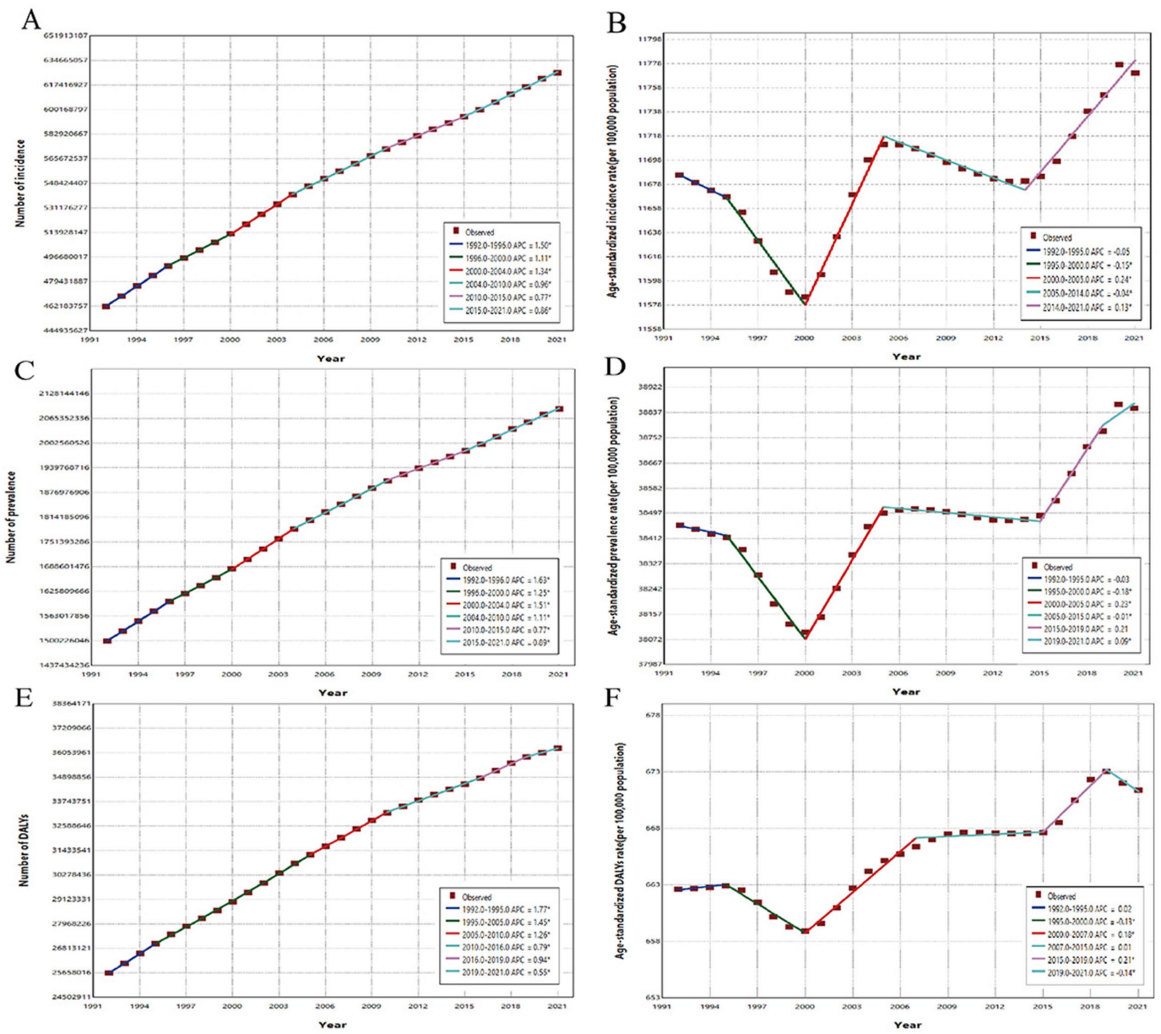
The joinpoint regression analysis of global incidence, prevalence and DALYs of Headache disorders in people under 50 years of age from 1992 to 2021. (A) The joinpoint regression analysis on the case number of incidence; (B) The joinpoint regression analysis on the ASR of incidence; (C) The joinpoint regression analysis on the case number of prevalence; (D) The joinpoint regression analysis on the ASR of prevalence; (E) The joinpoint regression analysis on the case number of DALYs; (F) The joinpoint regression analysis on the ASR of DALYs.

Globally, the case number of incidence, prevalence and DALYs of headache disorders in people under 50 years of age have increased significantly between 1992 and 2021. Surprisingly, the case number of incidence, prevalence and DALYs of headache disorders in 2021 reached 630 million, 2.09 billion and 36 million, respectively, an increase of 35, 39, and 41% compared to 1992. Trends in the burden of Headache disorders from 1992 to 2021 are shown in Figure 1. In terms of disease subtypes, the case number of the above indicators of migraine in 2021 is 80 million, 89 million, and 33 million, an increase of 32, 41, and 41% compared with 1992; The case number of TTH in 2021 will be 550 million, 1.49 billion, and 3 million, representing increases of 36, 39, and 41%. During these 30 years, the ASR of incidence, prevalence and DALYs in headache disorders showed an increasing trend, and AAPC was [0.03% (95%CI, 0.02–0.04%)], [0.04% (95%CI, 0.03–0.04%)] and [0.05% (95%CI, 0.03–0.06%)], respectively. Detailed information on age-standardized incidence, prevalence, and DALYs is shown in Table 1.

**Table 1:** Global incidence, prevalence, and DALYs of headache disorders and subtypes under 50s and their AAPCs, 1992–2021.

	1992		2021		AAPC (95%CI, 1992–2021)
	Number (95%CI)	ASR (95%CI)	Number (95%CI)	ASR (95%CI)	
Headache					
Incidence	462,183,757 (321,080,244–630,237,840)	11,685.75 (8,106.98–15,941.88)	626,110,007 (432,884,570–854,392,461)	11,770.03 (8,143.58–16,057.87)	0.03 (0.02–0.04)
Prevalence	1,500,226,047 (1,220,831,895–1,826,400,514)	38,457.80 (31,322.92–46,784.91)	2,089,478,831 (1,700,147,016–2,544,167,886)	38,850.75 (31,589.12–47,335.58)	0.04 (0.03–0.04)
DALYs	25,658,017 (4,100,705–57,115,856)	662.61 (109.66–1,467.28)	36,279,970 (5,935,161–80,671,095)	671.38 (108.12–1,496.71)	0.05 (0.03–0.06)
Migraine					
Incidence	61,048,600 (39,632,175–87,405,559)	1,518.30 (984.60–2,176.62)	80,780,907 (52,255,235–116,262,481)	1,538.18 (996.82–2,209.8)	0.04 (0.03–0.06)
Prevalence	632,420,167 (494,546,249–797,998,880)	16,280.12 (12,765.02–20,522.90)	893,075,504 (699,220,293–1,128,504,734)	16,548.15 (12,938.00–20,925.95)	0.06 (0.04–0.07)
DALYs	23,467,305 (2,728,696–54,482,011)	605.26 (72.96–1,397.83)	33,174,941 (3,971,158–76,727,550)	614.23 (72.35–1,424.17)	0.05 (0.03–0.07)
Tension-type headache					
Incidence	401,135,157 (260,144,344–567,539,054)	10,167.45 (6,593.01–14,376.50)	545,329,101 (352,764,930–770,171,731)	10,231.85 (6,619.55–14,458.20)	0.02 (0.01–0.04)
Prevalence	1,070,306,327 (743,727,086–1,442,703,981)	27,421.15 (19,060.71–36,926.97)	1,482,645,461 (1,027,917,604–2,004,980,827)	27,585.17 (19,119.60–37,324.14)	0.02 (0.01–0.04)
DALYs	2,190,712 (518,758–8,162,016)	57.34 (13.84–210.02)	3,105,029 (750,615–11,374,520)	57.16 (13.69–211.23)	−0.01 (−0.03 to 0.01)

### 3.2 Global trends of headache disorders by sex

From the perspective of gender, the case number and ASR of incidence, prevalence and DALYs are higher in females than males. In 2021, the case number of DALYs will be 22 million for females and 14 million for males. However, in the past 30 years, the above indicators have increased faster in males than in females, especially in DALYs [AAPC: 0.12%, (95%CI, 0.11–0.14%)]. While we are concerned about the burden of headache disorders in females, we cannot ignore the fact that its impact on males’ health is increasing (Table 2).

**Table 2:** Global incidence, prevalence and DALYs of headache disorders under 50s and their AAPCs by sex, 1992–2021.

		1992		2021		AAPC (95%CI, 1992–2021)
		Number (95%CI)	ASR (95%CI)	Number (95%CI)	ASR (95%CI)	
Incidence	Male	220,127,064 (150,401,353–302,775,858)	10,954.51 (7,477.85–15,073.09)	301,587,049 (205,644,977–414,621,942)	11,144.01 (7,602.66–15,319.16)	0.06 (0.05–0.08)
	Female	242,056,693 (169,336,978–328,193,537)	12,442.74 (8,691.96–16,875.84)	324,522,958 (226,415,749–441,107,259)	12,420.75 (8,673.87–16,876.84)	0 (−0.01 to 0.01)
Prevalence	Male	686,913,113 (545,073,936–852,829,526)	34,664.12 (27,528.15–43,009.29)	966,380,620 (769,073,080–1,203,377,543)	35,404.44 (28,160.50–44,106.64)	0.07 (0.07–0.08)
	Female	813,312,934 (672,613,087–973,368,858)	42,371.48 (35,077.07–50,669.94)	1,123,098,211 (928,317,873–1,346,011,838)	42,404.42 (35,021.01–50,868.04)	0.04 (0.03–0.04)
DALYs	Male	9,847,315 (1,730,620–21,898,824)	500.66 (91.15–1,107.63)	14,221,262 (2,510,327–31,439,769)	519.10 (90.39–1,150.12)	0.12 (0.11–0.14)
	Female	15,810,702 (2,371,570–35,413,207)	829.61 (128.82–1,848.55)	22,058,708 (3,430,734–49,199,335)	828.17 (126.51–1,852.58)	−0.01 (−0.02 to 0.01)

### 3.3 Global trends of headache disorders by age

In terms of age, the ASR of incidence in nine age groups peaked in youth aged 25–29, reaching 13,130.53 (95%CI, 9,374.20–17,856.70) per 100,000 population in 1992 and 13,454.64 (95%CI, 9,566.96–18,361.36) per 100,000 population in 2021. The ASR of prevalence is highest among middle-aged adults aged 30–34, reaching 48,505.80 (95%CI, 39,703.44–58,193.50) per 100,000 population in 1992 and 48,179.13 (95%CI, 39,328.29–57,839.44) per 100,000 population in 2021. The age group with the greatest impact on health is 40–44 years old, with ASR of DALYs of 902.58 (95%CI, 211.79–1,916.90) per 100,000 population in 1992 and 908.83 (95%CI, 204.92–1,955.68) per 100,000 population in 2021 (Table 3).

**Table 3:** Global incidence, prevalence and DALYs of headache disorders under 50s and their AAPCs by age, 1992–2021.

		1992		2021		AAPC (95%CI, 1992–2021)
		Number (95%CI)	ASR (95%CI)	Number (95%CI)	ASR (95%CI)	
Incidence	5–9	47,229,337 (33,446,371–65,175,702)	7,851.00 (5,559.84–10,834.25)	53,594,170 (37,877,124–73,784,257)	7,800.58 (5,512.98–10,739.23)	−0.02 (−0.04 to 0)
	10–14	70,258,417 (50,070,690–91,831,268)	12,782.24 (9,109.45–16,707.03)	84,985,591 (60,625,383–111,462,118)	12,748.43 (9,094.23–16,720.09)	−0.01 (−0.02 to 0.01)
	15–19	62,012,373 (43,178,054–84,487,642)	11,987.80 (8,346.88–16,332.57)	76,185,447 (52,385,223–104,348,308)	12,209.60 (8,395.34–16,723.02)	0.06 (0.03–0.09)
	20–24	60,861,742 (41,406,890–85,022,903)	12,156.61 (8,270.67–16,982.59)	75,235,597 (51,002,825–105,094,252)	12,598.94 (8,540.92–17,599.07)	0.12 (0.11–0.14)
	25–29	61,309,070 (43,770,007–83,376,501)	13,130.53 (9,374.20–17,856.70)	79,159,443 (56,168,881–108,027,827)	13,454.64 (9,546.96–18,361.36)	0.09 (0.06–0.11)
	30–34	52,178,376 (35,947,362–70,973,017)	13,080.07 (9,011.28–17,791.51)	78,080,217 (53,788,196–105,651,104)	12,916.92 (8,898.26–17,478.01)	−0.05 (−0.07 to −0.03)
	35–39	45,676,739 (31,235,577–62,722,699)	12,398.86 (8,478.83–17,025.95)	69,711,598 (47,522,560–96,230,809)	12,429.28 (8,473.07–17,157.54)	0.01 (0–0.02)
	40–44	37,220,761 (24,741,436–51,303,109)	11,979.37 (7,962.94–16,511.73)	60,243,108 (40,342,516–82,339,708)	12,042.58 (8,064.46–16,459.69)	0.03 (0.01–0.04)
	45–49	25,436,942 (17,283,858–35,344,998)	10,442.46 (7,095.43–14,509.94)	48,914,838 (33,171,862–67,454,078)	10,330.38 (7,005.60–14,245.71)	−0.04 (−0.07 to −0.01)
Prevalence	5–9	56,921,273 (40,895,981–76,223,573)	9,462.10 (6,798.20–12,670.75)	64,661,865 (46,406,948–86,518,582)	9,411.47 (6,754.49–12,592.70)	−0.02 (−0.04 to 0)
	10–14	184,111,191 (148,672,577–226,052,119)	33,495.69 (27,048.27–41,126.08)	222,953,339 (179,294,276–274,190,448)	33,444.54 (26,895.38–41,130.46)	0 (−0.02 to 0.01)
	15–19	210,321,204 (166,262,004–257,015,760)	40,657.84 (32,140.62–49,684.51)	258,209,281 (204,214,037–315,613,783)	41,381.03 (32,727.66–50,580.76)	0.06 (0.04–0.08)
	20–24	215,653,763 (179,063,659–262,943,215)	43,074.99 (35,766.43–52,520.65)	265,896,686 (220,347,801–325,160,311)	44,527.01 (36,899.41–54,451.29)	0.12 (0.1–0.13)
	25–29	211,636,939 (176,304,663–254,875,136)	45,326.18 (37,759.08–54,586.48)	273,959,209 (227,496,903–331,041,097)	46,564.52 (38,667.38–56,266.66)	0.1 (0.08–0.13)
	30–34	193,496,944 (158,383,007–232,142,668)	48,505.80 (39,703.44–58,193.50)	291,233,266 (237,731,687–349,627,944)	48,179.13 (39,328.29–57,839.44)	−0.03 (−0.05 to −0.01)
	35–39	174,416,868 (140,959,305–209,485,143)	47,345.10 (38,263.11–56,864.31)	267,499,942 (216,888,598–321,956,653)	47,694.08 (38,670.30–57,403.48)	0.03 (0.01–0.04)
	40–44	146,245,533 (120,921,818–178,254,602)	47,068.61 (38,918.26–57,370.62)	236,842,850 (195,457,718–289,200,904)	47,344.82 (39,071.95–57,811.18)	0.03 (0.01–0.05)
	45–49	107,422,332 (89,368,881–129,408,299)	44,099.36 (36,688.00–53,125.11)	208,222,394 (172,309,046–250,858,163)	43,974.73 (36,390.15–52,979.03)	−0.01 (−0.04 to 0.02)
DALYs	5–9	552,286 (18,867–1,439,285)	91.81 (3.14–239.25)	639,873 (21,485–1,693,511)	93.13 (3.13–246.49)	0.05 (0–0.09)
	10–14	2,534,622 (160,402–6,029,314)	461.13 (29.18–1,096.92)	3,107,094 (195,142–7,393,463)	466.09 (29.27–1,109.07)	0.04 (0–0.07)
	15–19	3,523,794 (414,830–8,319,514)	681.20 (80.19–1,608.27)	4,355,498 (500,396–10,395,457)	698.02 (80.19–1,665.99)	0.08 (0.06–0.11)
	20–24	3,798,412 (508,142–8,718,219)	758.70 (101.50–1,741.39)	4,660,549 (615,989–10,675,888)	780.45 (103.15–1,787.78)	0.1 (0.08–0.12)
	25–29	3,721,011 (515,931–8,427,585)	796.93 (110.50–1,804.93)	4,791,002 (652,507–10,833,551)	814.32 (110.91–1,841.37)	0.08 (0.06–0.09)
	30–34	3,408,730 (582,209–7,246,495)	854.50 (145.95–1,816.55)	5,158,630 (874,441–11,025,012)	853.40 (144.66–1,823.88)	0 (−0.01 to 0.01)
	35–39	3,244,867 (725,849–6,689,803)	880.81 (197.03–1,815.93)	4,989,592 (1,076,080–10,429,412)	889.62 (191.86–1,859.52)	0.03 (0.02–0.05)
	40–44	2,804,395 (658,037–5,955,945)	902.58 (211.79–1,916.90)	4,546,422 (1,025,135–9,783,288)	908.83 (204.92–1,955.68)	0.02 (0.01–0.04)
	45–49	2,069,900 (516,437–4,289,695)	849.74 (212.01–1,761.02)	4,031,311 (973,985–8,441,512)	851.38 (205.70–1,782.77)	0.01 (−0.01 to 0.03)

### 3.4 Global trends of headache disorders by SDI

**Figure 2:**
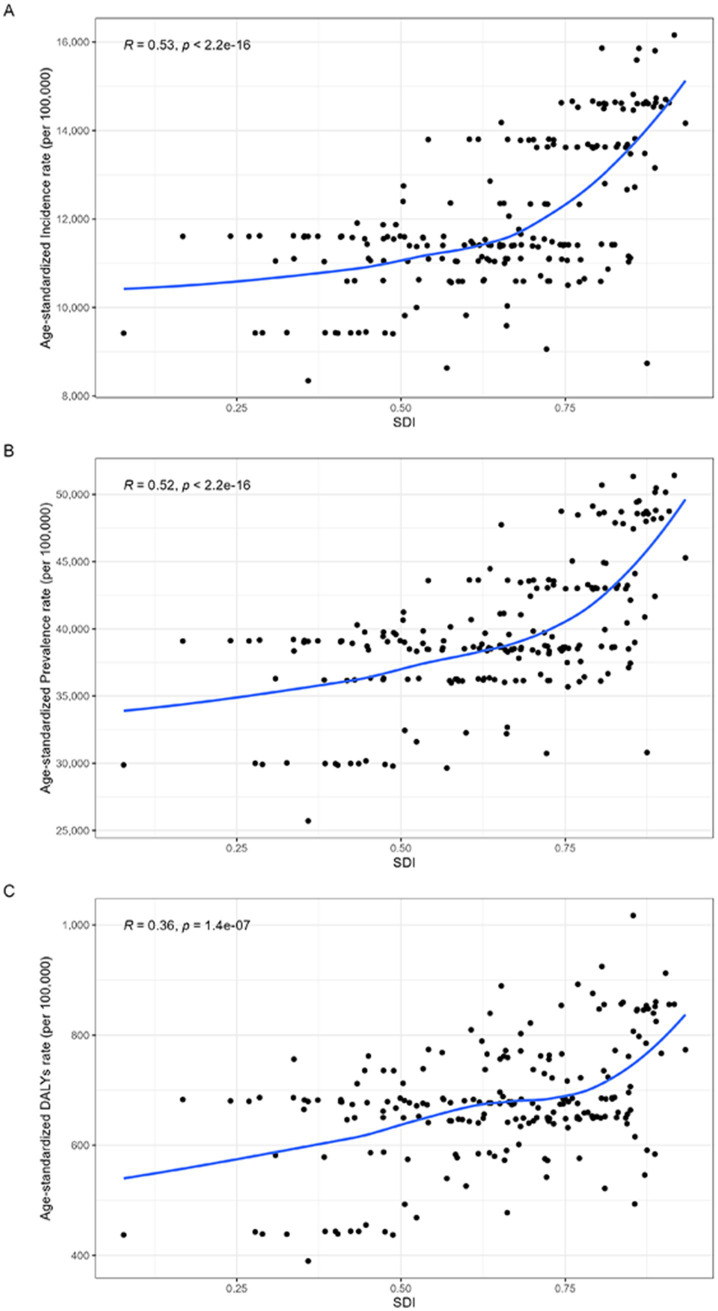
The ASR of headache disorders burden for people under 50 years of age in 2021 according to the SDI. (A) Relationship between the ASR of incidence rate and SDI; (B) Relationship between the ASR of prevalence rate and SDI; (C) Relationship between the ASR of DALYs rate and SDI.

Through scatter plots, the correlation between SDI and the ASR of headache disorders was depicted in detail. Spearman correlation analysis showed a positive correlation between ASR and SDI in patients under 50 years of age with headache disorders globally (*R* = 0.53 and *p* < 0.001, *R* = 0.52 and *p* < 0.001, *R* = 0.36 and *p* < 0.001, respectively, Figure 2). From the perspective of SDI, the highest ASR for incidence, prevalence and DALYs were observed in high SDI, with an incidence of 14,095.94 (9,548.09–19,357.44) per 100,000 population in 2021, with an prevalence of 40,567.16 (95%CI, 36,830.58–54,420.67) per 100,000 population, DALYs were 741.59 (95%CI, 124.01–1,637.27) per 100,000 population. However, in 2021, the highest case number of middle SDI was observed; Meanwhile, the growth trend of middle SDI during these three decades is the largest, namely [AAPC, 0.20% (95%CI, 0.18–0.22%)], [AAPC, 0.20% (95%CI, 0.18–0.22%)] and [AAPC, 0.18% (95%CI, 0.15–0.21%)], respectively Table 4. According to GBD 2021, we can find that the country with the highest SDI is Switzerland and the country with the lowest SDI is Somalia. We visualized the number of cases and ASR of DALYs in the two countries over the past 30 years, and found that the ASR in both countries remained relatively stable, while the number of cases in Somalia showed a substantial increase from 21,432 (95%CI, 4,622–47,796) in 1992 increased to 61,652 (95%CI, 13,528–138,025) in 2021; In Switzerland, the number of cases increased more slowly, from 36,888 (95%CI, 6,468–81,537) in 1,992–41,546 (95%CI, 7,493–91,116) in 2021 (Supplementary Figure 1).

**Table 4:** Global incidence, prevalence and DALYs of headache disorders under 50 years of age and their AAPCs by SDI, 1992–2021.

		1992		2021		AAPC (95%CI, 1992–2021)
		Number (95%CI)	ASR (95%CI)	Number (95%CI)	ASR (95%CI)	
Incidence	High SDI	85,440,432 (57,868,274–117,605,796)	14,208.21 (9,647.09–19,556.28)	88,459,912 (59,666,721–121,537,816)	14,095.94 (9,548.09–19,357.44)	−0.03 (−0.03 to −0.02)
	High-middle SDI	86,252,721 (59,616,730–117,949,588)	11,162.00 (7,714.30–15,258.82)	90,247,559 (62,195,376–123,391,231)	11,340.74 (7,836.29–15,496.71)	0.06 (0.04–0.07)
	Middle SDI	143,472,552 (100,349,507–195,094,323)	10,727.61 (7,492.36–14,592.52)	187,498,829 (30,180,518–255,236,790)	11,359.03 (7,897.94–15,454.86)	0.2 (0.18–0.22)
	Low-middle SDI	105,954,442 (73,625,733–144,034,973)	12,052.56 (8,348.74–16,410.33)	169,465,608 (117,380,507–231,198,155)	12,018.61 (8,321.09–16,397.85)	−0.01 (−0.01 to −0.01)
	Low SDI	40,612,719 (27,975,157–55,788,918)	11,063.32 (7,594.29–15,217.50)	89,946,551 (61,937,369–123,838,683)	10,959.78 (7,525.79–15,109.99)	−0.03 (−0.04 to −0.02)
Prevalence	High SDI	282,200,156 (231,644,789–339,367,784)	45,420.20 (37,162.15–54,740.25)	296,890,038 (243,478,925–357,466,964)	45,067.16 (36,830.58–54,420.67)	−0.03 (−0.04 to −0.02)
	High-middle SDI	285,420,301 (231,984,454–346,979,206)	36,512.99 (29,650.75–44,429.16)	310,686,319 (252,436,103–377,752,566)	37,312.47 (30,204.90–45,503.00)	0.08 (0.06–0.1)
	Middle SDI	471,813,753 (384,702,157–574,040,998)	35,858.56 (29,258.23–43,606.96)	640,364,035 (523,117,768–777,410,843)	37,933.81 (30,952.48–46,101.46)	0.2 (0.18–0.22)
	Low-middle SDI	334,547,193 (271,215,448–409,413,248)	39,805.60 (32,368.03–48,594.91)	558,063,324 (453,592,627–680,913,986)	39,731.03 (32,301.58–48,464.43)	−0.01 (−0.01 to 0)
	Low SDI	124,772,407 (99,083,301–155,462,078)	36,135.13 (28,850.82–44,833.84)	281,816,150 (224,154,059–350,493,418)	35,875.58 (28,659.55–44,478.82)	−0.02 (−0.04 to −0.01)
DALYs	High SDI	4,678,918 (820,861–10,259,527)	740.34 (124.72–1,634.94)	5,011,043 (907,710–10,923,141)	741.59 (124.01–1,637.27)	0 (0–0.01)
	High-middle SDI	4,961,884 (976,295–10,808,978)	631.78 (124.60–1,376.03)	5,541,564 (1,119,125–11,989,797)	647.33 (121.59–1,418.51)	0.08 (0.08–0.09)
	Middle SDI	8,347,040 (1,220,274–18,805,088)	639.98 (98.02–1,431.53)	11,497,798 (1,794,077–25,603,178)	674.50 (101.72–1,510.15)	0.18 (0.15–0.21)
	Low-middle SDI	5,651,935 (757,404–12,838,163)	685.70 (99.18–1,541.78)	9,608,113 (1,366,981–21,652,102)	686.12 (99.66–1,542.08)	0 (−0.01 to 0.01)
	Low SDI	1,993,489 (314,637–4,504,579)	592.71 (102.27–1,320.99)	4,592,457 (734,180–10,376,593)	596.13 (102.71–1,330.86)	0.02 (0.01–0.03)

### 3.5 Global trends of headache disorders by country

**Figure 3:**
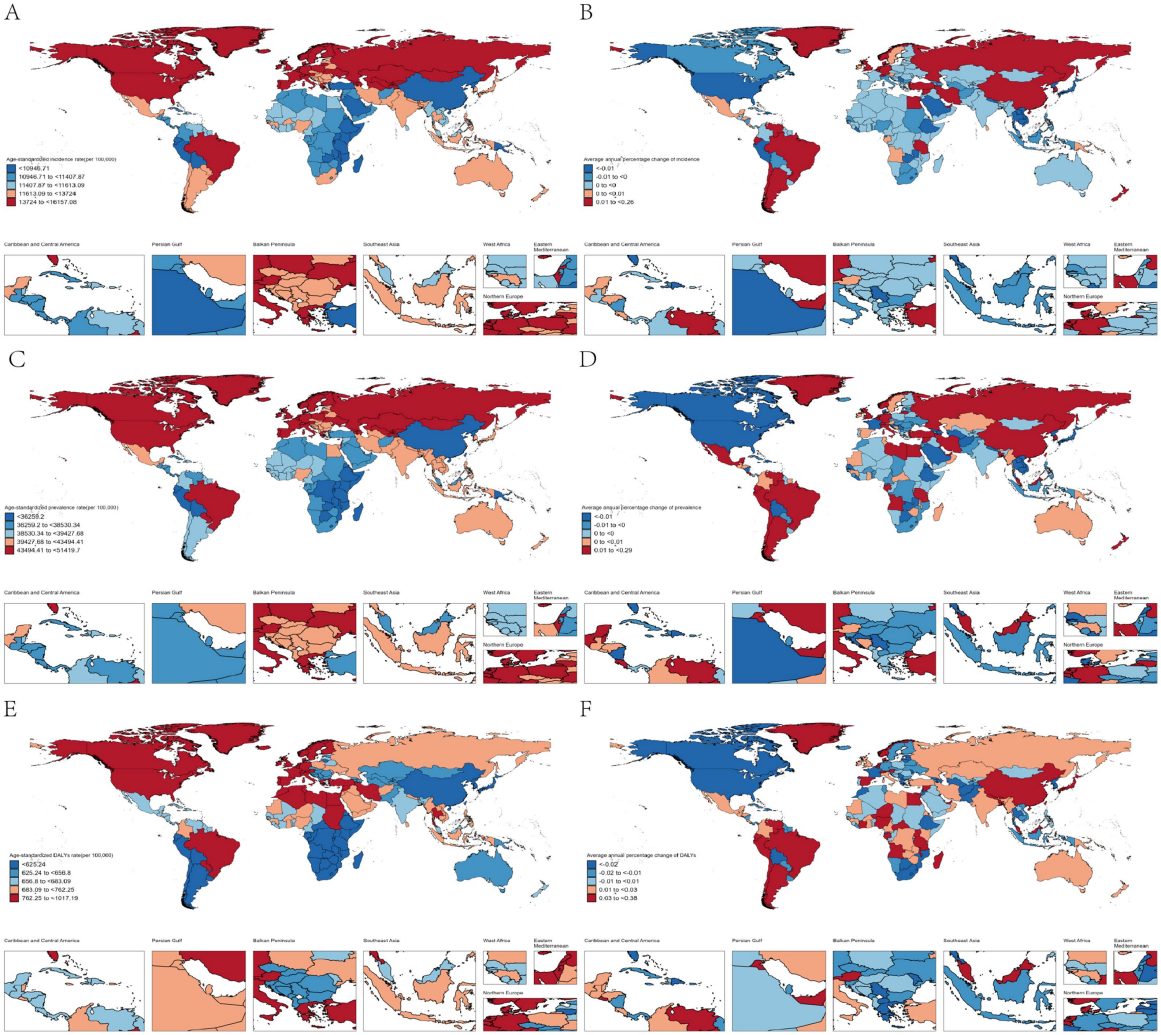
Analysis of trends in the burden of headache disorders among people under 50 years of age in 204 countries and regions. (A) The ASR of incidence in 2021; (B) The AAPC of incidence from 1992 to 2021; (C) The ASR of prevalence in 2021; (D) The AAPC of prevalence from 1992 to 2021; (E) The ASR of DALYs in 2021; (F) The AAPC of DALYs from 1992 to 2021.

Due to differences in genetic background, climatic and socio-economic conditions, lifestyle, other disease profiles, and general health status, the burden of headaches can vary considerably in different countries of the world. We looked at the current burden of headache disorders in people under 50 years of age across countries. At the national level, Norway has the highest ASR of incidence and prevalence in 2021, at 16,157 (95%CI, 10,960–22,020) per 100,000 population and 51,420 (95%CI, 42,876–60,919) per 100,000 population; the highest ASR of DALYs were in Belgium, at 1,017 (95%CI, 124–2,293) per 100,000 population. Over the past three decades from 1992–2021, overall trends in disease burden vary widely among countries. China had the highest increase in the incidence of headache disorders [AAPC, 0.26% (95%CI, 0.19–0.32%)]. The highest increases in prevalence and DALYs occurred in Singapore, [AAPC, 0.29% (95%CI, 0.27–0.31%)] and [AAPC, 0.38% (95%CI, 0.33–0.43%), respectively] (Figure 3).

### 3.6 Global trends in subtypes of headache disorders by country

**Figure 4:**
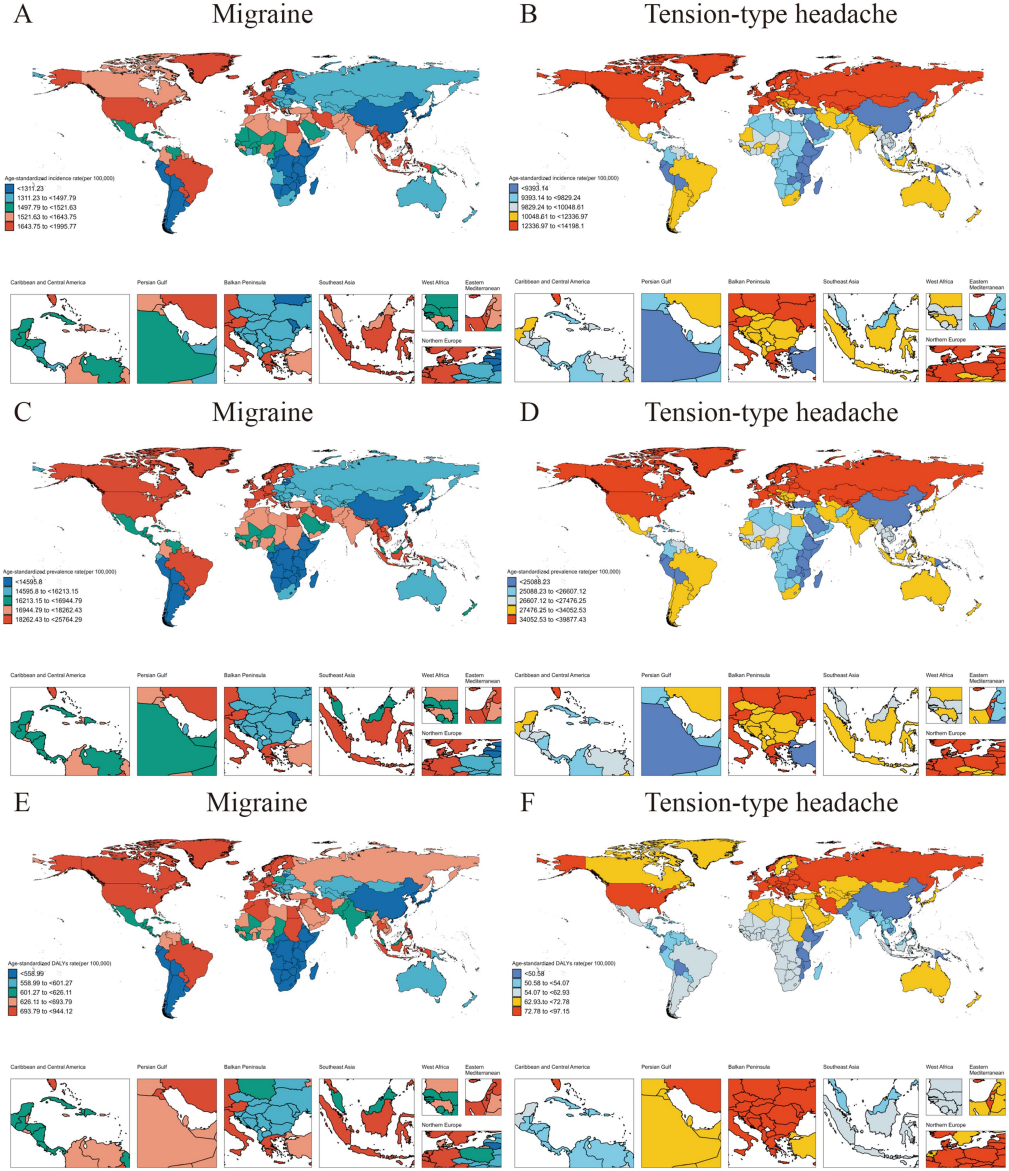
The burden of migraine and TTH in 2021 for people under 50 years of age was assessed in 204 countries and regions. (A) The ASR of migraine incidence in 2021; (B) The ASR of TTH incidence in 2021; (C) The ASR of migraine prevalence in 2021; (D) The ASR of TTH prevalence in 2021; (E) The ASR of migraine DALYs in 2021; (F) The ASR of TTH DALYs in 2021.

According to the GBD 2021, headache disorders can be divided into migraine and TTH. Next, the ASR of the current incidence, prevalence and DALYs of these two subtypes are analyzed at the national level. In migraine, the country with the highest ASR of the above three indicators is Belgium, 1996 (95%CI, 1,186–3,040) per 100,000 population, 25,764 (95%CI, 19,792–32,949) per 100,000 population and 944 (95%CI, 83–2,224) per 100,000 population, respectively; The country with the highest ASR of incidence and prevalence of TTH is Norway, were 14,198 (95%CI, 9,017–19,994) per 100,000 population and 39,877 (95%CI, 29,128–51,781) per 100,000 population; The highest ASR of DALYs was found in the Russian Federation, which had a DALYs of 97 (95%CI, 27–315) per 100,000 population. Detailed information on the ASR of migraine and TTH for 204 countries and territories is available in Figure 4.

### 3.7 Global trends of headache disorders by regions and age

**Figure 5:**
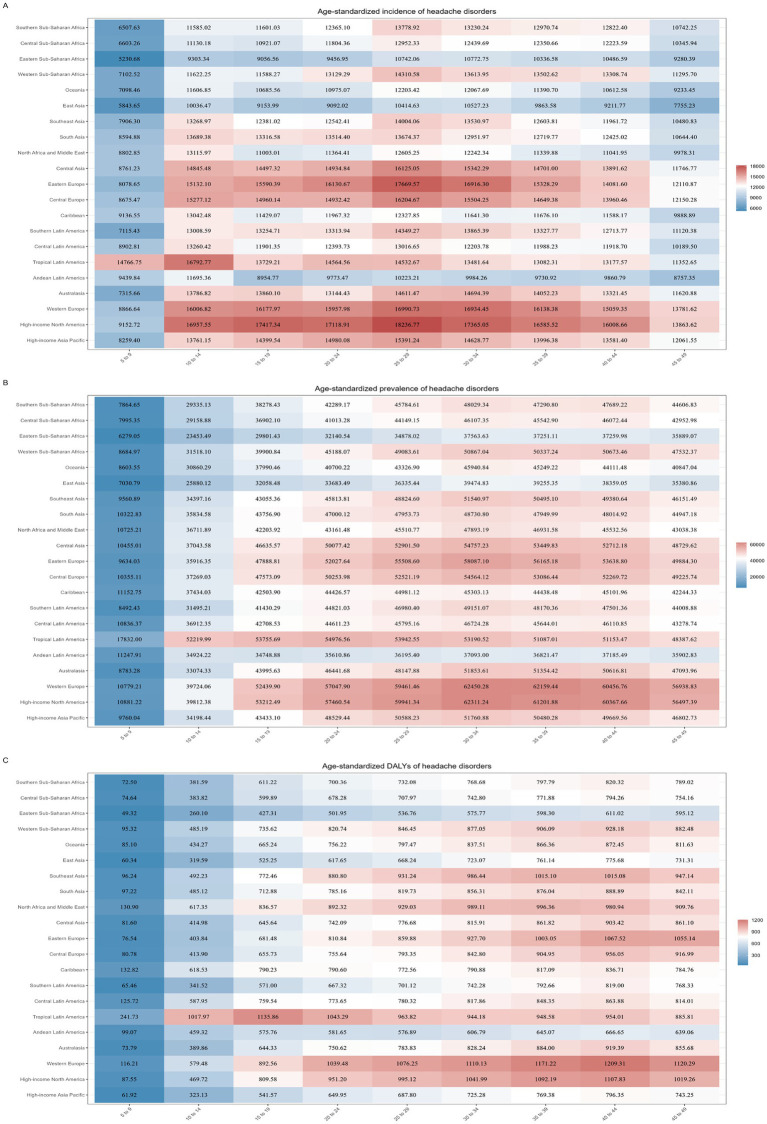
Assess the burden of headache disorders in people under 50 years of age in 2021 by regions and age. (A) The ASR of incidence of headache disorders in people under 50 years of age by regions and age; (B) The ASR of prevalence of headache disorders in people under 50 years of age by regions and age; (C) The ASR of DALYs of headache disorders in people under 50 years of age by regions and age.

After age standardization, the data of headache disorders among people under 50 years old were disaggregated by regions and age, and it was found that the incidence of headache disorders in High-income North America, Western Europe, Eastern Europe, Central Europe and Central Asia was higher than that in other regions. The highest ASR of incidence was found in the 25–29 age group in High-income North America, reaching 18,237 (95%CI, 12,505–25,066) per 100,000 population; A similar phenomenon was observed in prevalence, with the highest ASR of prevalence observed in the 30–34 year age group in Western Europe, reaching 62,450 (95%CI, 51,981–73,490) per 100,000 population; The highest ASR of DALYs were found in the 40–44 age group in Western Europe, at 1,209 (95%CI, 284–2,584) per 100,000 population, and surprisingly, DALYs among adolescents 15–19 in Tropical Latin America were also high, reaching 1,136 (95%CI, 75–2,732) per 100,000 population (Figure 5).

### 3.8 Cross-country analysis of inequality in headache disorders

**Figure 6:**
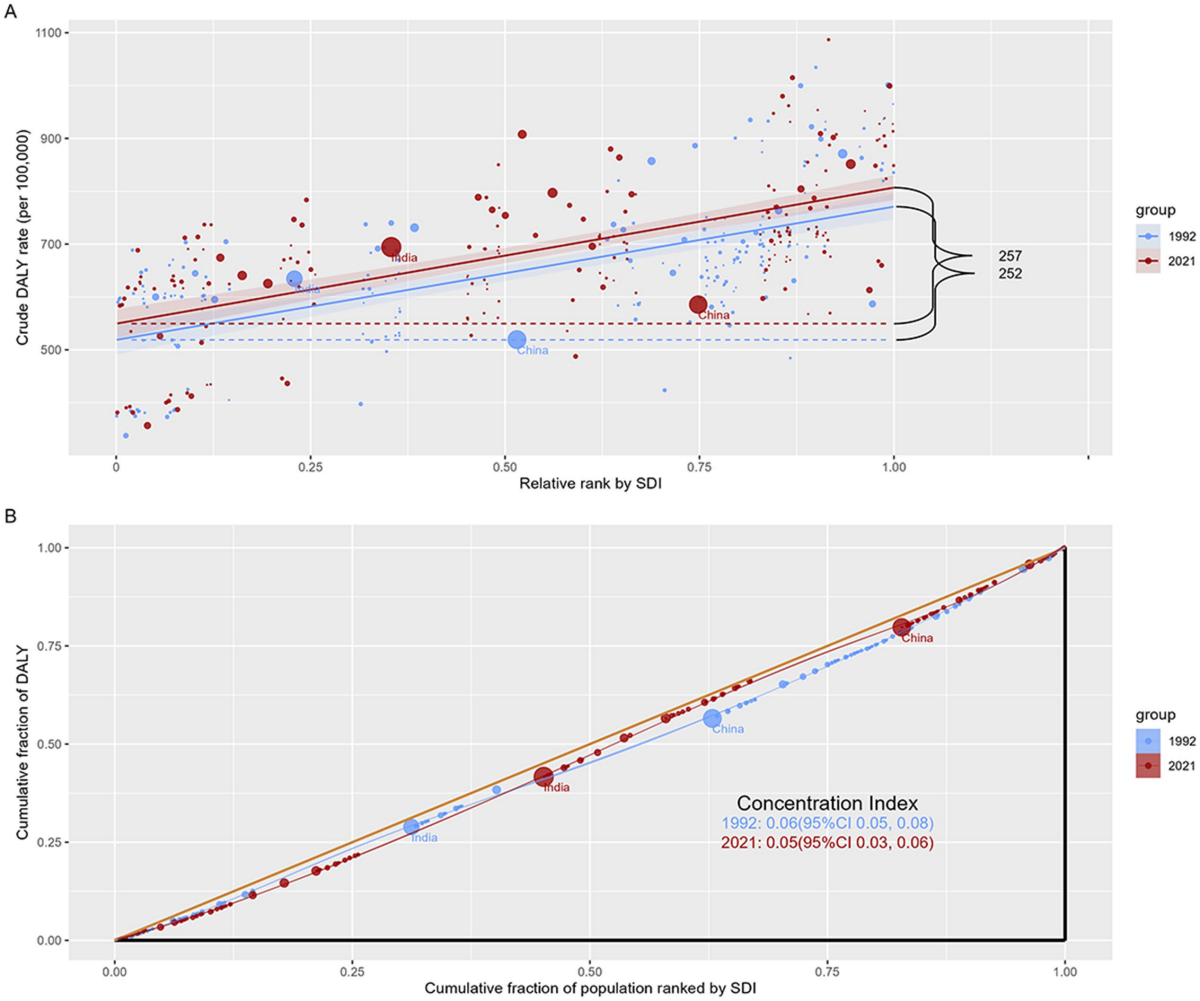
Analysis of SDI-related health inequality in headache disorders in under 50s. (A) Slope index of inequality: absolute inequality of SDI-related DALYs; (B) Concentration index of inequality: relative inequality of SDI-related DALYs.

In order to better clarify the health inequities of headache disorders among countries with different SDI, we studied the absolute and relative inequalities related to DALYs and SDI in headache disorders burden, and found that these inequalities increased over time. As shown in Figure 6A, the Slope index is positive, indicating that DALYs is more common in countries with high SDI. In 1992, the difference in DALY crude rate between the highest SDI and the lowest SDI was 252 (95%CI, 209–295) per 100,000 population, slightly increased to 257 (95%CI, 215–300) per 100,000 population in 2021. As shown in Figure 6B, Concentration index is 0.06 (95%CI, 0.05–0.08) in 1992 and 0.05 (95%CI, 0.03–0.06) in 2021, both of which are positive. This indicates that DALYs are more concentrated in countries with high SDI. From 1992 to 2021, although the Concentration index has declined (from 0.06 to 0.05), it is still positive, indicating that this unhealthy concentration, while perhaps diminishing, still exists. This may reflect health inequalities on a global scale and suggest the need for further research and action to reduce such inequalities (Figure 6).

### 3.9 Prediction of the global burden of headache disorders by 2046

**Figure 7:**
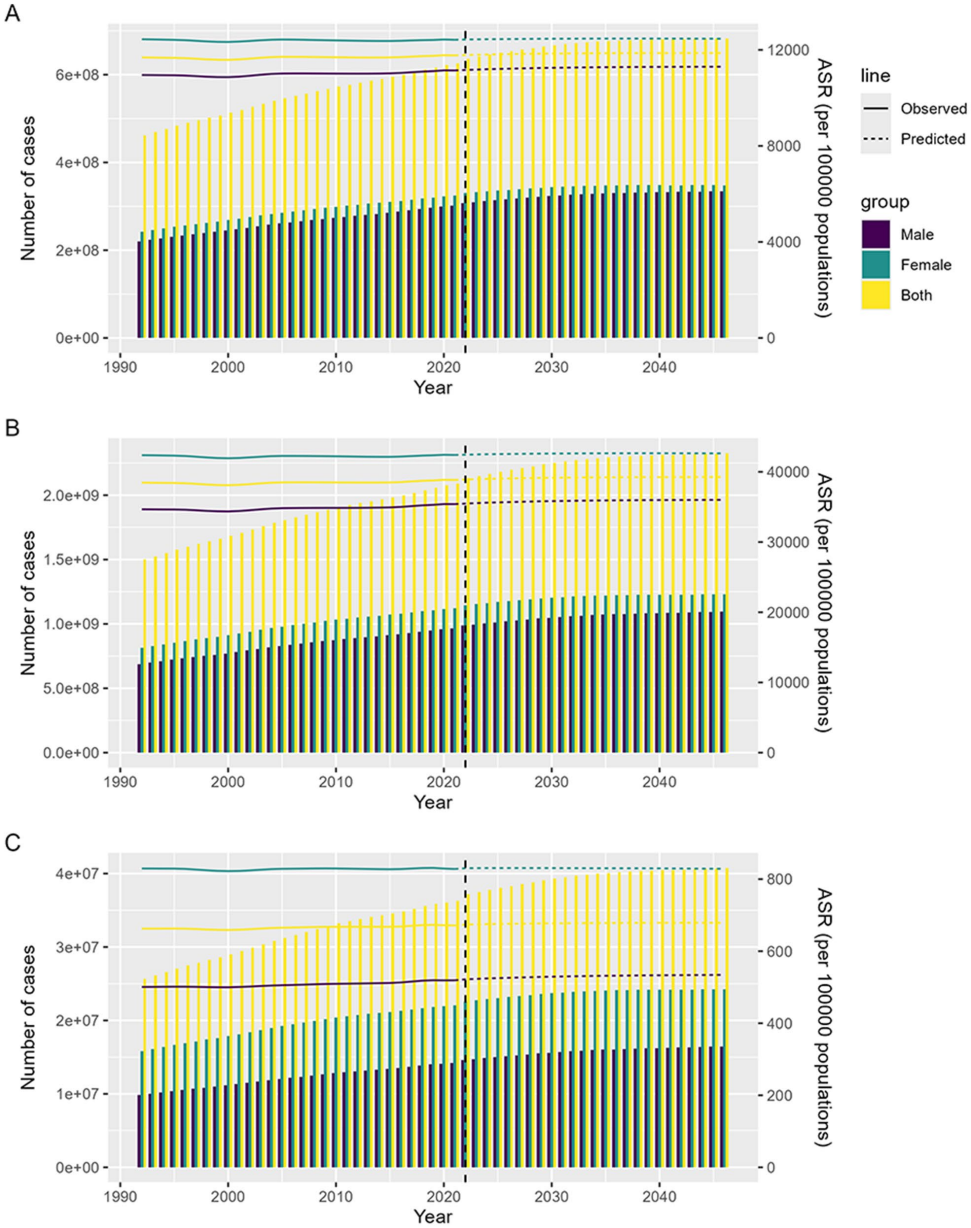
Prediction of the global burden of headache disorders in under 50s from 2022 to 2046. (A) The predicted number of cases of headache disorders incidence by 2046; (B) The predicted number of cases of headache disorders prevalence by 2046; (C) The predicted number of cases of headache disorders DALYs by 2046.

To better understand the future global burden of headache disorders among people under 50 years of age, we forecast the case number and ASR of incidence, prevalence and DALYs for headache disorders in the next 25 years based on an age-period-cohort model. From 2022 to 2046, the case number of incidence, prevalence and DALYs of headache disorders are expected to increase globally, but ASR for all three measures will remain relatively stable. Specifically, by 2046, the case number is expected to increase from 630 million in 2021 to 680 million; prevalence will increase from 2.09 billion to 2.33 billion; DALYs will increase from 36 million to nearly 41 million (Figure 7). These predictions suggest that the burden of headache disorders in people under 50 years of age will persist long into the future. Although some medical interventions and health promotion measures may be taken, the headache problem has not been effectively addressed. This means that although there may be some improvement in the short term, in the long term, the health burden of headache disorders in this age group remains high and requires further attention and new response measures.

## 4 Discussion

This study obtained the latest data on the incidence, prevalence and DALYs of headache disorders in people under 50 years of age from 1992 to 2021 from the recently updated GBD 2021, and further provides a comprehensive assessment based on trends, disaggregation, inequality and predictive analysis. In this study, we drew the following important conclusions: the burden of headache disorders among people under 50 years of age has remained high and has not improved significantly over the past 30 years. At the same time, the distribution and trend of the burden show unique characteristics that are closely related to factors such as gender, age and regional social development level. Compared to 1992, the number of people under the age of 50 suffering from headache disorders increased by 160 million in 2021, with TTH accounting for 87% of the total. Although the number of cases of TTH is far greater than that of migraine, it is clear that migraine have a greater impact on people’s health and their financial burden. Overall, there was little change in the ASR of headache disorders and no significant increase. It shows that the global campaign to combat headache disorders—Lifting the burden, co-sponsored by the World Health Organization (WHO), the International Headache Society (IHS), the World Headache Federation (WHF) and the European Headache Federation (EHF), has played a positive role to some extent (21). Over the past two decades, headache disorders have gained widespread public health attention as a result of the “Lighting the burden” movement. The campaign effectively raised public awareness and awareness of the burden of headache disease and, in the process of implementation, effectively improved research capacity in low—and middle-income countries. It also gave us a broader picture of the burden of headaches, informing all iterations of GBD since 2010 (21). Despite advances in treatment, those advances have not been effective in reaching people with headaches, so the overall burden has not substantially improved, reflecting the continuing profound public health and socio-economic impact of the disease (22). According to the WHO, migraine alone is responsible for an estimated 25 million lost working or school days in the UK each year; This financial loss can be equivalent to TTH and chronic daily headache combined (23). Associated with this are long-standing and unaddressed barriers to care around the world, creating a huge gap between the demand for care and the actual supply. These medical deficiencies can be traced back to inadequate education. This lack of understanding has led to the treatment of pain not being prioritized, and there is clear evidence that this is the wrong approach. Such neglect is potentially harmful from a public health and economic point of view, as it can lead to significant financial pressures. Educational inadequacies hinder public awareness and thus allow this important message to be ignored (21).

Previous studies have shown that headache is more common in female, who are more likely to experience headache during periods such as menstruation, pregnancy, and menopause due to changes in hormone levels (24, 25). In addition, studies have shown that obesity may increase the risk of migraine in female (26). Our results also confirm that the burden of disease is greater in female than in male (27), but the burden of disease is increasing faster in male than in female. At the same time, males generally suffer from greater work and life stress, excessive alcohol consumption, and changes in testosterone levels can all be trigger factors for headaches (28–30). The above also reminds us that in the process of preventing and treating headache disorders, we should not only pay attention to the prevention and treatment of female headache, but also should not ignore its development in male groups. The incidence of headache peaks in young people aged 25–29 years, who face great stress in life and work, unhealthy lifestyles and prolonged use of electronic devices can trigger headaches (31). In addition, a recent large cohort study showed that migraine usually begins before the age of 18, not adulthood as previously thought (32). Our study also included the latest data on headache disorders in children and adolescents, which further supports this view by showing that a significant number of children and adolescents already have headaches. Therefore, in the prevention and treatment of diseases, children and adolescents should be a group of special concern. We also observed a positive correlation between the burden of headache disorders and SDI in people under 50 years of age, with higher levels of ASR in countries and regions with high SDI. This may be related to the greater work and life pressure faced by people in countries and regions with high SDI, as well as environmental pollution caused by economic development (33). This is also supported by trend analysis at the country level, with Norway and Belgium, as highly developed countries, having the highest ASR of incidence, prevalence and DALYs; China has developed rapidly in the past 30 years, so the incidence has increased the most during this period. From 2022 to 2046, the case number of incidence, prevalence, and DALYs in people under 50 years of age will continue to increase, while the ASR remains stable. This suggests that headache disorders will remain a major public health problem for people under 50 for the next 25 years. At present and in the future, the burden of headache disorders among people under 50 remains significant, while current health policies and prevention and control strategies have not shown significant effectiveness. The WHO reports that many people suffering from headache disorders do not receive effective treatment. For example, in the United States and the United Kingdom, only half of the patients identified as having migraine sought medical attention for their headaches in the past 12 months, and only two-thirds received the correct diagnosis. Most people only receive treatment with over-the-counter medications (23). In summary, this study suggests that headache disorders impose a significant health burden on people under 50 years of age, and that global progress in controlling headache burden over the past 30 years has been unsatisfactory. Headache disorders are not only a nuisance for some people, but also cause widespread suffering for patients and their families, and impose significant economic costs on society. Specifically, headaches may affect an individual’s daily life and work productivity, making it difficult for the patient to perform school, work, or social activities. This constant feeling of discomfort often leads to low mood and poor concentration, which further worsens the symptoms (7). Therefore, countries around the world must implement targeted interventions against the most prominent risk factors for headache in their respective contexts to prevent or slow down this growth, thereby improving the health of their populations and alleviating pressure on healthcare systems and national budgets.

It is important to note that the whole burden of headaches is entirely due to the non-fatal health outcome: in other words, years lived with disability (YLDs) match with DALYs. The YLDs for headache disorders is the average time the patient spends with that type of headache multiplied by the relevant disability weight (DW). The determination of headache DW was made through population and Internet surveys based on non-professional descriptions. But the definition of DW is still very partial in its hyper simplified description (9). Therefore, some researchers have proposed the use of patient-reported outcome measures (PROMs) to comprehensively analyze the disability of headache disorders (9). PROMs shifts from a disease-centered perspective to a patient-centered perspective, and closely analyzes the life experience of headache patients with reduced ability to perform daily activities. By translating subjective symptoms into objective scores, PROMs makes the assessment of headaches more quantifiable and standardized, making it easier for doctors and researchers to analyze and compare. But they have shown significant limitations in real-world clinical practice. The reliability of monitoring PROMS patients is not as good as expected, and choosing an ideal set of short, non-invasive tests to provide useful information is very difficult. In PROMs, patients may give different answers to the same question a few minutes apart (34). While PROMs still has some limitations, it is undeniable that it will enable the objectification of headache-related disability and inform future iterations of the GBD study (9).

## 5 Conclusion

This study comprehensively analyzed and evaluated the burden and prevalence of headache disorders in people under 50 years of age. Globally, the disease remains a serious public health problem for this age group. Over the past 30 years, with social development and lifestyle changes, the burden of disease has remained high and has not been significantly improved, which puts a lot of pressure on individual health and social economy. According to our results, the health damage caused by migraine is much greater than that caused by TTH. This finding suggests that the focus of medical resource allocation and mechanism research should be tilted toward migraine to ensure that patients have access to more effective treatment and management. In addition, our analysis showed that headache disorders were significantly more damaging to health in areas with high SDI and in middle-aged people aged 35–44 years than in other age groups. This phenomenon may be related to the greater occupational pressure, life burden and physiological changes faced by individuals in this age group. In areas with high SDI, people are under greater psychological and physical stress due to high economic activity, which further exacerbates the frequency and severity of headache attacks. Therefore, when formulating public health policies, special attention needs to be paid to these specific populations so that targeted interventions can be taken to improve their overall health. This study reveals the distribution characteristics of headache in geographical, age and sex dimensions, providing important epidemiological evidence for health policy makers. We hope that by rationally planning and allocating medical resources, optimizing management models, and conducting effective preventive work, the physical and mental burdens on individual patients can be alleviated, and the cost pressure faced by the entire society can be reduced. We look forward to the emergence of more targeted scientific solutions in the future, which can provide more reliable protection for alleviating headache problems among people under the age of 50.

## Data Availability

The datasets presented in this study can be found in online repositories. The names of the repository/repositories and accession number(s) can be found at: http://vizhub.healthdata.org/gbd-results/.

## Footnotes

^1^ http://vizhub.healthdata.org/gbd-results/
