# Supplementary material for: Headache disorders: a persistent public health challenge for the under 50s
Source: Front Neurol. 2024 Oct 23;15:1501749. doi: 10.3389/fneur.2024.1501749 (PMC11538006; doi:10.3389/fneur.2024.1501749)
Supplement: Supplementary file 1 [file Data_Sheet_1.PDF]

Supplementary Figure 1：

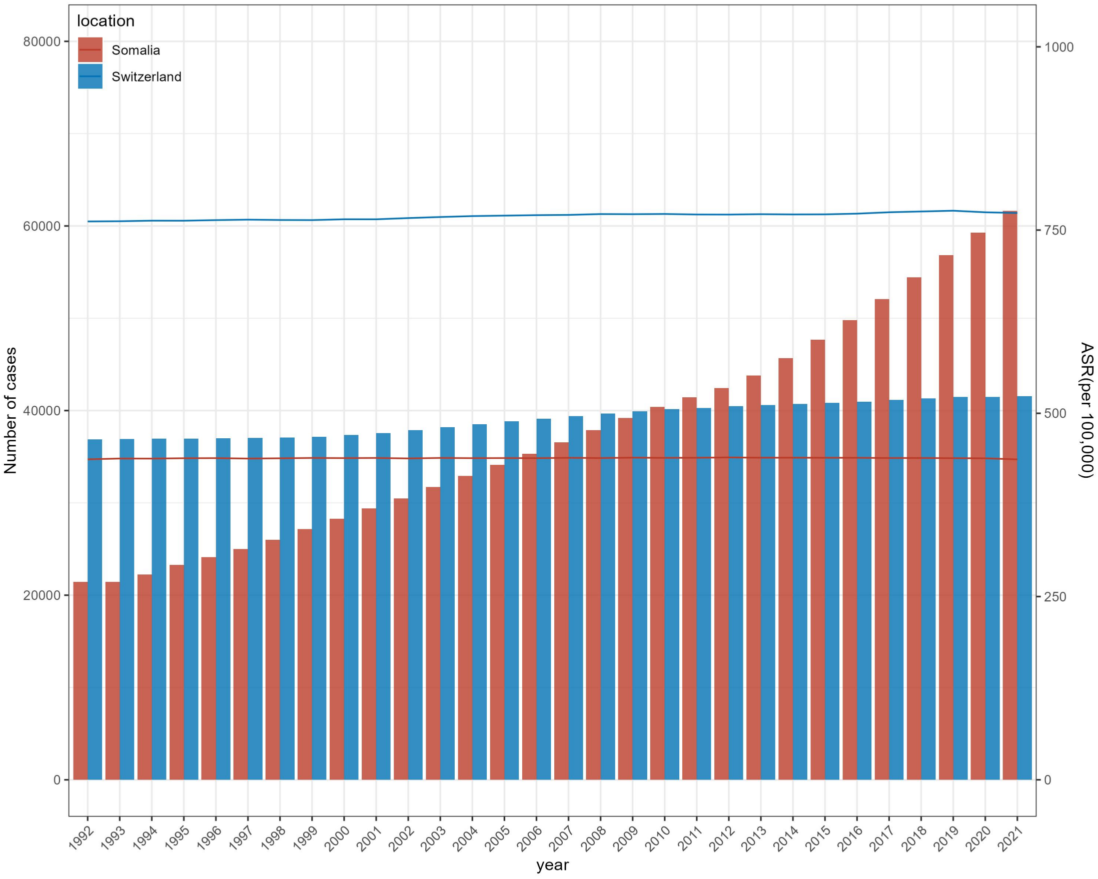

Supplementary Figure 1: The number of cases and ASR of DALYs for headache disorders under 50s in Somalia and Switzerland from 1992 to 2021; Blue:Switzerland;Red:Somalia;Bar:Number of cases;Line:ASR.
